# Mechanical instability as a signature of viscoelastic decoupling at the tumor–brain interface

**DOI:** 10.1016/j.bioadv.2026.214758

**Published:** 2026-02-02

**Authors:** Jan Saip Aunan-Diop, Ancuta Ioana Friismose, Emi Hojo, Ziying Yin, Bo Halle, Frederik Harbo, Bo Mussmann, Frantz Rom Poulsen

**Affiliations:** a Department of Neurosurgery, Odense University Hospital, Denmark; b Brain Research Interdisciplinary Guided Excellence (BRIDGE), University Of Southern Denmark, Denmark; c Department of Radiology, Odense University Hospital, Denmark; d Research Unit of Radiology, Department of Clinical Research, University of Southern Denmark, Odense, Denmark; e Department of Radiology, Mayo Clinic, Rochester, MN, United States of America

**Keywords:** Magnetic resonance elastography, Tumor–brain interface, Viscoelasticity, Mechanical instability, Glioblastoma, Meningioma

## Abstract

Brain tumors alter the viscoelastic equilibrium of surrounding tissue, but how these changes shape the mechanics of tumor–brain coupling remains unclear. This study introduces mechanical instability mapping, a voxelwise measure of imbalance between elastic storage and viscous dissipation derived from magnetic resonance elastography (MRE). Twenty-eight patients (15 meningiomas, 13 glioblastomas) were analyzed using standardized 3 T MRE and tumor segmentation. Quantitative descriptors of instability topology—including skeleton length and branch-point densities, and radial persistence (radial-AUC)—were compared across WHO I, WHO II, and glioblastoma groups. Glioblastomas showed diffuse, branched instability fields with significantly higher skeleton and branch-point densities and lower radial-AUC compared with WHO I meningiomas, which exhibited compact, radially coherent patterns. Group-average probability maps indicated a transition from coherent to fragmented instability with increasing malignancy. These findings demonstrate that peritumoral mechanical topology reflects the degree of viscoelastic coupling at the tumor–brain interface. Instability mapping thereby extends conventional stiffness-based MRE metrics, offering a quantitative framework for assessing interface integrity and heterogeneity that may aid in elasticity-guided treatment strategies and biomechanical phenotyping of brain tumors.

## 1. Introduction

The brain is an exceptionally soft, viscoelastic organ whose structural integrity reflects a finely balanced interplay between its solid and fluid components. Foundational biophysics and biomechanics work shows that neural tissue exhibits pronounced nonlinearity, viscoelasticity, and poroelastic fluid–solid coupling across scales (from axons and extracellular matrix (ECM) to whole-brain response) [1-3]. When a tumor develops within this environment, it introduces marked mechanical heterogeneity—changing how stresses are transmitted, dissipated, and redistributed in the surrounding parenchyma. Such changes can influence tumor progression and the practical mechanics of surgical resection. Conceptually, a tumor and its microenvironment form a coupled viscoelastic system in which contrasts in elasticity, viscosity, and interfacial adhesion govern the deformation field at multiple scales [4,5].

Tumor expansion perturbs local microarchitecture, compresses and remodels vasculature, and reorganizes the ECM. The ECM itself can stiffen or undergo architectural changes that alter stress propagation and mechanotransduction, shaping cell behavior and invasion [4,6,7]. In gliomas, for example, abnormal ECM composition and mechanics have been linked to altered cell migration and diffuse infiltration [8]. At mesoscopic scales, heterogeneity in ECM viscoelasticity and fluid–matrix coupling lead to local contrasts in stress relaxation and energy dissipation. These contrasts can guide deformation along preferential paths, such that portions of the tumor–brain interface maintain coherent mechanical coupling while others decouple irregularly [5].

Clinically, the consequences of this mechanical coupling are well recognized, as differences in stiffness and interface cohesion manifest directly in operative handling and surgical and treatment outcomes [9-13]. Low grade meningiomas are frequently firm and well circumscribed, presenting with a separable plane at the brain surface, whereas glioblastomas (GBMs) are more infiltrative and lack a distinct dissection plane. These observations align with differences in tumor–parenchyma mechanics reported in imaging and intraoperative series. Magnetic resonance elastography (MRE) studies, including those performed by this group, show that meningiomas are stiffer, while GBMs are softer and exhibit higher damping/viscous behavior than normal tissue [14-18]. Spatial variations in viscoelasticity are a feature of brain tumors, with mechanical heterogeneity often most pronounced near the tumor–parenchyma boundary [8,17,18].

From a physical standpoint, the tumor–brain interface behaves as a boundary between two viscoelastic continua with contrasting mechanical properties. When this contrast exceeds a critical threshold, the interface may lose coherence and develop localized perturbations or slip bands, analogous to those observed in soft-matter systems under differential strain [4,19-21]. Similar interfacial instabilities are well established in physics. The Saffman–Taylor instability in viscous flows describes the branching of an interface when a low-viscosity fluid displaces a more viscous one [20,22,23]. In solids, Biot's surface instability (1963) and subsequent theories of viscoelastic bifurcation demonstrate how competition between elastic storage and viscous dissipation gives rise to wrinkling, shear bands, and other localized deformation patterns [21,24]. In biological soft tissues, this same principle manifests through extracellular matrix remodeling and cytoskeletal reorganization under mechanical stress [8,25].Collectively, these frameworks converge on a unifying principle: mechanical instability arises when the equilibrium between stored and dissipated energy is disrupted. Consequently, the morphology of high-instability regions observed in MRE maps may encode the topology of mechanical coupling and failure.

The aim of this study is to determine whether the spatial organization of peritumoral mechanical instability differs systematically between benign and malignant intracranial tumors, and whether these patterns offer a biomechanical explanation for distinct clinical behavior. We tested the following hypotheses:

- WHO I meningiomas exhibit confined, symmetric peritumoral instability consistent with stable elastic coupling and a well-defined interface;
- WHO II meningiomas show locally expanded or irregular instability, indicating partial interface disruption;
- GBMs display diffuse, branched instability extending into surrounding parenchyma, consistent with infiltrative and more dissipative coupling.

## 2. Methods

### 2.1. Study design and participants

The study was conducted in accordance with the Declaration of Helsinki and approved by the Regional Committee on Health Research Ethics for the Region of Southern Denmark (IDs: S-20190105, S-20220055). All participants provided written informed consent prior to inclusion. Patients with suspected intracranial meningiomas scheduled for surgical resection at the Department of Neurosurgery, Odense University Hospital, Denmark, were consecutively recruited. For comparison, patients with newly diagnosed glioblastoma (GBM) were included from an independent prospective study applying the same MRE acquisition protocol [26].

### 2.2. Image acquisition

MRE was performed on a 3 T system (Achieva, Philips Healthcare, The Netherlands) using a 16-channel head coil. The examination included a contrast-enhanced T1-weighted (T1W) gradient-echo sequence after intravenous gadobutrol (Gadovist 1 mmol/mL; Bayer, Germany) at 0.1 mL/kg. Shear waves at 60 Hz were generated using a pneumatic driver (Resoundant Inc., USA) positioned beneath the head, operated at approximately 20% of the manufacturer's maximum amplitude.

Displacement fields were acquired using a single-shot spin-echo echo-planar MRE sequence (TR/TE = 4800/67 ms; field of view = 240 × 240 mm^2^; SENSE = 3; 48 slices; slice thickness = 3 mm; voxel = 3 mm^3^; 8 phase offsets).

Elastograms were reconstructed using a direct inversion algorithm to yield maps of the storage modulus G′ (elasticity), loss modulus G″ (viscous damping), and the magnitude ∣G*∣ [26].

All maps were rigidly co-registered and resampled to the ∣G*∣ grid using 3D Slicer (v5.6) with visual verification [27].

### 2.3. Tumor segmentation and region definition

For meningiomas, tumor segmentation was performed semiautomatically on contrast-enhanced T1W images in 3D Slicer. Boundaries were outlined manually (JSA, 3 years' experience), refined by region-growing, and inspected manually. Ten tumor were resegmented after 12 weeks to assess intra-observer reliability.

For glioblastomas, ROIs were delineated independently by two observers—a research assistant (AIF, 3 years) and a neuroradiologist (FSH, 9 years)—including only contrast-enhancing solid tumor while excluding necrosis and cystic areas.

T1-based brain masks were generated using SynthStrip, refined to parenchyma masks, and eroded by 1 voxel for meningiomas to avoid partial-volume effects at the cortical surface [28].

### 2.4. Definition of tumor rim and peritumoral shell

The rim region (r) was defined as the 1-voxel-thick layer immediately outside the tumor within the parenchyma.

The peritumoral shell (p) was defined as voxels located 0–6 mm from the tumor boundary, excluding voxels within 2 mm of the skull. The rim region corresponds to the tumor–brain interface transition zone, consistent with prior imaging and histopathological literature [29,30]. Distances were computed in physical mm using an anisotropy-aware Euclidean distance transform.

These definitions were applied identically to meningiomas and GBMs.

### 2.5. Mechanical instability field

Mechanical instability was modeled as a voxel-wise scalar field I(x) describing the local imbalance between elastic storage and viscous dissipation relative to the rim reference.

Within the rim (r), the median loss modulus $G^{\prime \prime }(r,\text{med})$ and median damping ratio (tanδ)(r,med) were computed after all zero or negative value voxels were excluded from the ROIs.

For each voxel x in the peritumoral shell (p), instability was defined as:

$$I(x)=ln\left(\frac{G^{\prime \prime }(x)}{G_{rmed}^{\prime }}\right)\cdot (tan\delta (x)-{\left(tan\delta \right)}_{rmed})$$

where $G^{\prime \prime }(x)$ and δ(x) are the local loss modulus and damping ratio.

The logarithmic term quantifies the viscous contrast, and the phase-lag term captures local differences in dissipation.

Resulting maps were masked to parenchyma, saved as NIfTI volumes, and visualized alongside G′, G″, and T1W images with overlaid contours.

### 2.6. Instability-derived metrics

From each subject's peritumoral domain, a series of quantitative descriptors were derived to summarize the magnitude, spatial extent, and topology of mechanical instability. The median and 95th percentile of the instability field I(x) were calculated, together with the fraction of voxels exceeding the thresholds I>0.02 and I>0.05. The cutoff for defining instability (I>0.02) was chosen from the pooled histogram of all cases at the point where background fluctuations gave way to coherent peritumoral structures. The entropy of the histogram (32 bins, range 0–0.4) quantified mechanical heterogeneity within the peritumoral region.

To characterize the radial organization of instability, profiles I(r) were computed in 2-mm concentric shells extending up to 12 mm from the tumor boundary only including parenchyma. The area under this curve (AUC) represented the persistence of mechanical perturbation with distance. The integrated survival function S(t)=P(I>t)up to I=0.30 provided a complementary measure of the overall extent of instability, reported as the Tail-AUC. Radial profiling beyond 6 mm was used for this characterization only, and not for defining the peritumoral analysis domain.

Morphological properties of the instability field were analyzed on the axial slice containing the maximal tumor area. Thresholded maps (I>0.02) were used to compute the $isoperimetricratio(IPR)=4\pi A∕P^{2}$, reflecting boundary irregularity, and convexity, defined as the ratio between the region area and the area of its convex hull $\left(A∕A_{convex}\right)$.

To capture the internal topology of mechanically unstable regions, each thresholded map was converted into a binary mask and skeletonized using an 8-connected morphological algorithm implemented in *scikit-image*. The resulting one-voxel-wide skeleton preserved the medial axis of each unstable component. Skeleton length density was computed as the total number of skeleton voxels divided by the number of voxels in the unstable region, representing the normalized total length of unstable filaments per unit area. Branch-point density was defined as the proportion of skeleton voxels with three or more neighboring skeleton voxels (in 8-connectivity), quantifying the degree of branching and fragmentation of the instability network.

Together, these parameters describe the tortuosity, fragmentation, and spatial persistence of the peritumoral mechanical field. Per-case values were subsequently aggregated by diagnostic group (WHO I, WHO II, GBM) for statistical comparison.

### 2.7. Group-average instability and skeleton maps

To visualize group-level patterns, each subject's instability map I(x) was cropped to a 120 × 120 mm physical field centered on the axial slice containing the maximal tumor cross-section and resampled to a uniform 256 × 256 grid in physical space. Voxels exceeding the instability threshold (I>0.02) were binarized, skeletonized using 8-connectivity, and branch points identified where three or more skeleton segments converged.

For each group g∈{WHOI,WHOII,GBM}, occupancy $O_{i}$, skeleton $S_{i}$, and branch $B_{i}$ maps were averaged voxelwise over n_g cases to yield group-probability maps:

$$P_{g}^{occ}(u,v)=(1∕n_{g})\sum_{i\in g}O_{i}(u,v)P_{g}^{skel}(u,v)=(1∕n_{g})\sum_{i\in g}S_{i}(u,v)P_{g}^{br}(u,v)\;\;=(1∕n_{g})\sum_{i\in g}B_{i}(u,v)$$

Group maps were displayed with fixed ranges (0–1.0, 0–0.8, 0–0.6) for direct comparison.

The resulting figure illustrates the probability and geometric complexity of unstable regions in WHO I, WHO II, and GBM tumors.

### 2.8. Robustness analyses

To ensure that group differences in instability topology were not driven by arbitrary parameter choices, we performed a series of robustness checks. First, we varied the instability cutoff over a plausible range (I>0.015,0.020,0.025,0.030). For each threshold, per-case topology metrics—radial persistence (radial-AUC), skeleton length density, and branch density—were recomputed within the 0–6 mm peritumoral shell. Second, to assess sensitivity to pixel-scale irregularities, skeletonization was repeated after a light binary morphology opening (1.5 mm in-plane).

We quantified the fraction of negative instability values $\left(p_{\text{ne}}g\right)$ per case to indicate how much of the peritumoral field behaved elastically (mechanically stable) versus dissipatively. Fourth, to examine potential size bias, we evaluated correlations between topology metrics (e.g., radial-AUC) and tumor size proxies such as shell voxel count or tumor area.

Finally, to verify that spatial topology was not confounded by tumor scale, we generated *radius-normalized group probability maps* in which each tumor was rescaled to a common effective radius (R* = 20 mm) prior to averaging.

To relate local topology to bulk mechanical contrast, we computed rim–shell differences in storage modulus and damping $\Delta G^{\prime }=G_{\text{rim}}^{\prime }-G_{\text{shell}}^{\prime }$; $\Delta tan\delta ={\left(tan\delta \right)}_{\text{rim}}-{\left(tan\delta \right)}_{\text{shell}}$, merged these with per-case topology metrics, and evaluated associations using Spearman correlation.

Per-case metrics were summarized by their median across thresholds to avoid single-cutoff dependence. Given the small WHO II cohort (*n* = 5), we performed a bootstrap sensitivity analysis (case-resampling with replacement within each group; 10,000 iterations) for the primary topology metrics (radial-AUC, skeleton length density, branch-point density) to quantify the probability that WHO II occupies an ‘intermediate’ position (WHO I < WHO II < GBM or WHO I > WHO II > GBM, depending on metric direction) and to obtain 95% bootstrap confidence intervals for median between-group differences.

### 2.9. Statistical analyses

Computation and statistical analyses were performed in Python (v3.11) using pandas, scipy.stats, and matplotlib.

Distributions of continuous variables were assessed visually by histograms and Q−Q plots. Because most parameters showed non-normal distributions, non-parametric tests were applied throughout.

For each instability-derived metric (tail-AUC, proportion above 0.02 and 0.05, entropy, isoperimetric ratio, convexity, skeleton branch-point density, skeleton length density, and radial AUC), group differences were evaluated using the Kruskal–Wallis test.

If the overall test reached significance (*p* < 0.05), pairwise comparisons between WHO I, WHO II, and GBM were conducted with two-sided Mann–Whitney *U* tests.

Multiple testing was controlled using the Benjamini–Hochberg false discovery rate (FDR) procedure applied separately across all pairwise contrasts and metrics.

The adjusted q-value threshold for significance was q < 0.05.

Effect sizes for pairwise comparisons were expressed as Cliff's delta (δ), interpreted as negligible ∣δ∣<0.147, small (0.147–0.33), medium (0.33–0.474), or large (>0.474).

The Hodges–Lehmann estimator (HL) of the median difference was additionally reported to indicate the magnitude and direction of the shift between groups.

## 3. Results

### 3.1. Cohort

**Fig. 1.**
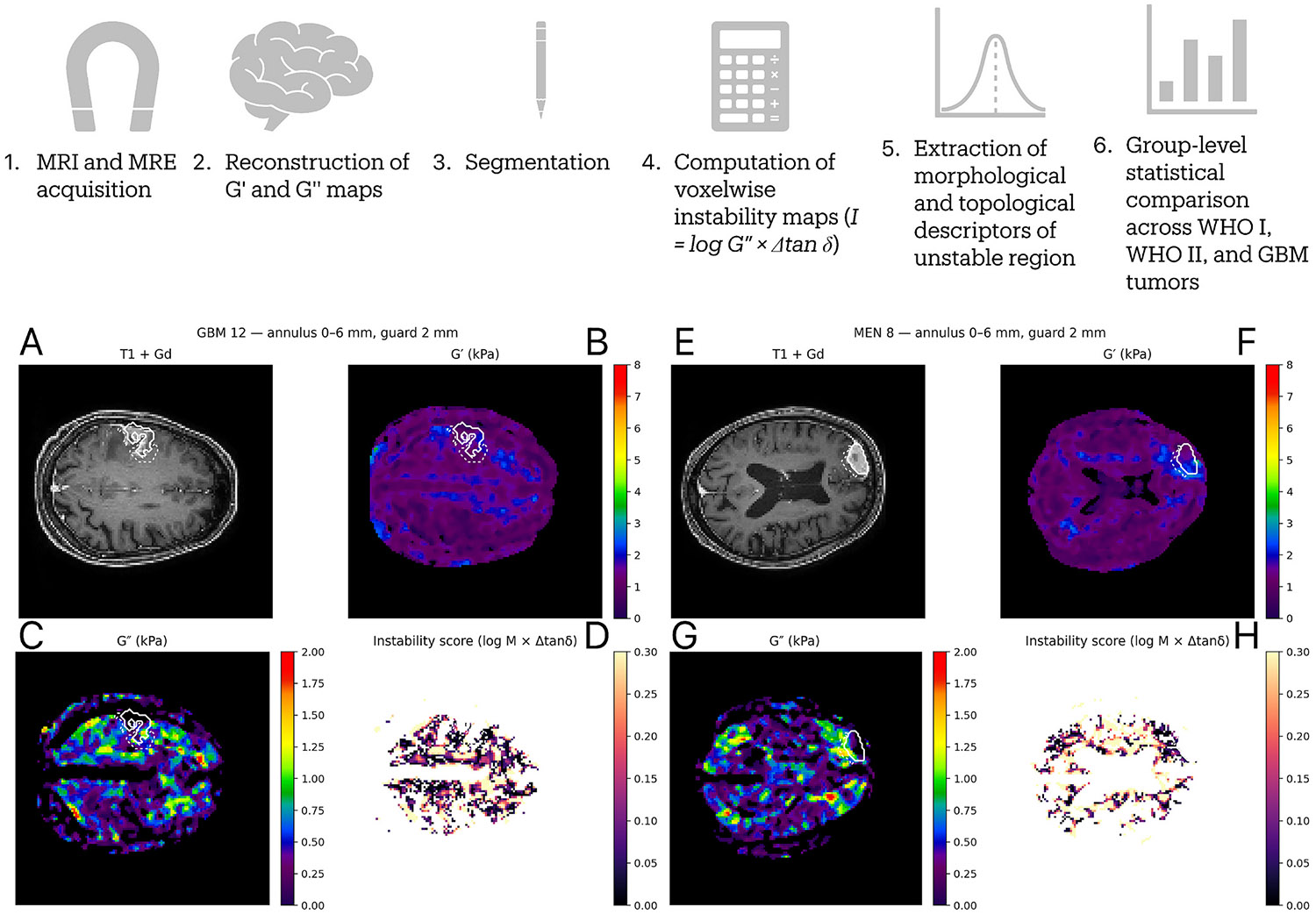
Overview of the analytical workflow and representative examples of mechanical instability mapping in glioblastoma and meningioma. The top panel summarizes the analytical pipeline, beginning with MRI and magnetic resonance elastography (MRE) acquisition, reconstruction of viscoelastic maps of storage (G′) and loss (G″) moduli, and tumor segmentation on contrast-enhanced T1-weighted images. Voxelwise mechanical instability was computed within the peritumoral annulus (0–6 mm, guard 2 mm) as the instability index (I), followed by extraction of morphological and topological descriptors for group-level comparison across WHO I, WHO II, and glioblastoma (GBM) cohorts. The lower panels show representative examples from a GBM (A–D) and a WHO I meningioma (E–H), displaying the contrast-enhanced T1-weighted image, G′ and G″ maps, and the derived instability score. White contours delineate the tumor and analysis shell.

Twenty-eight tumors were analyzed: fifteen meningiomas (10 WHO grade I, 5 WHO grade II) and thirteen GBMs (Table 1). All datasets met predefined image quality and segmentation criteria, and all cases were successfully processed through the voxelwise instability pipeline. Fig. 1 displays the analytical flow.

### 3.2. Groupwise distributions of instability metrics

Descriptive metrics summarizing the topology of peritumoral instability are reported in Table 2. Groupwise differences were observed across diagnostic categories. Median skeleton branch-point density increased progressively from 0.015 in WHO I meningiomas to 0.029 in WHO II and 0.151 in GBMs, indicating an increasing number of local bifurcations within the unstable region. Median skeleton length density followed a similar pattern, increasing from 0.126 (WHO I) to 0.324 (WHO II) and 0.326 (GBM), suggesting a greater cumulative length of unstable structures per unit area in higher-grade lesions. Conversely, radial-AUC, which quantifies the persistence of instability with distance from the tumor margin, was highest in WHO I meningiomas (1.193), lower in GBM (0.714), and lowest in WHO II (0.546). No substantial group differences were noted for convexity or isoperimetric ratio, with median convexity values ranging from 0.50 to 0.54 and IPR values from 8.7 to 18.1 across groups.

### 3.3. Kruskal–Wallis group effects

**Fig. 2.**
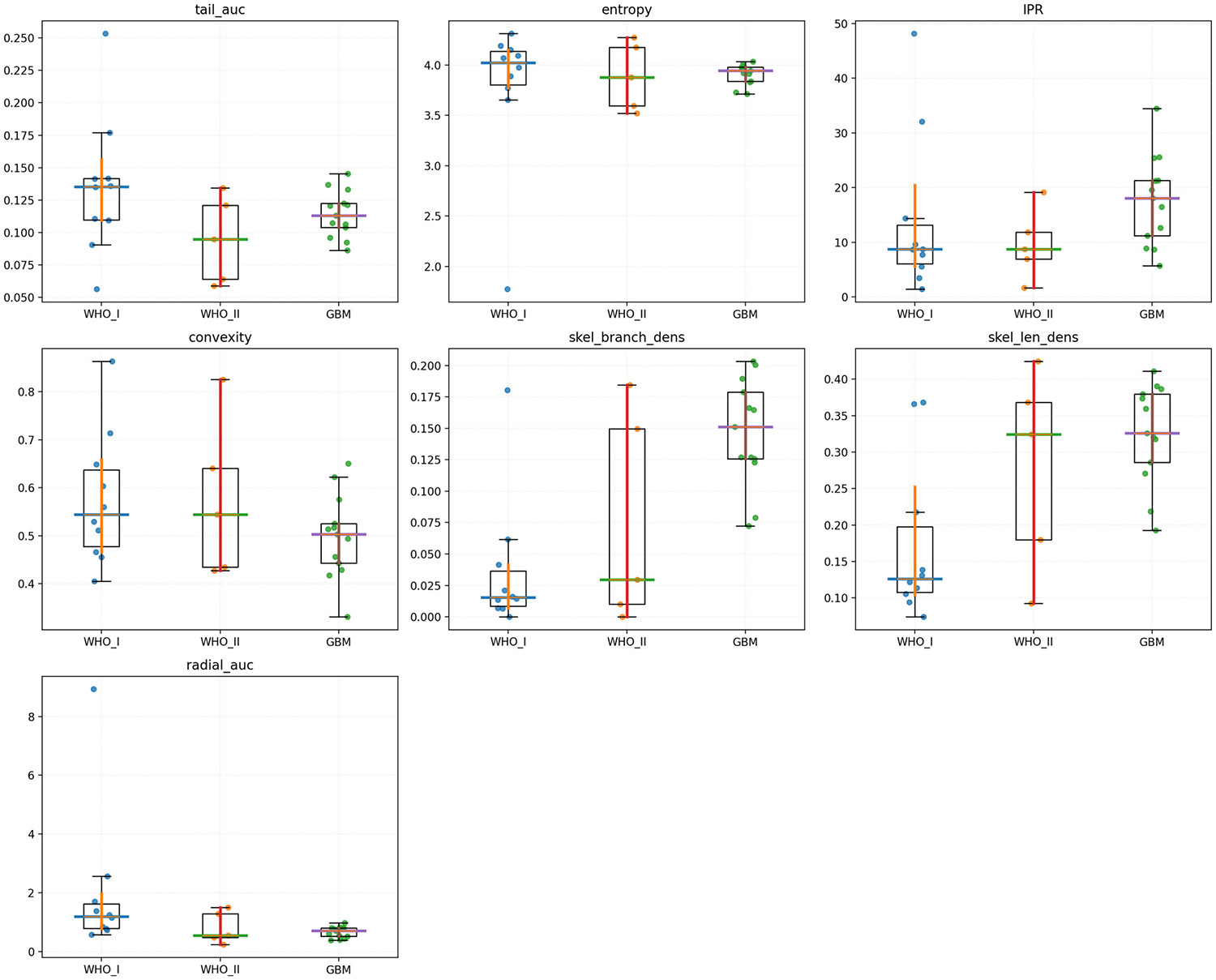
Groupwise distributions of instability-derived metrics. Box-and-swarm plots show metrics summarizing peritumoral mechanical instability in WHO I meningiomas (blue), WHO II meningiomas (orange), and glioblastomas (green). Horizontal bars indicate medians with bootstrap 95% CIs. Significant overall group effects (Kruskal–Wallis) were observed for skeleton branch-point density, skeleton length density, and radial-AUC (all *p* < 0.05); other metrics were not significant. Pairwise Mann–Whitney tests with BH-FDR correction identified WHO I vs GBM differences for radial-AUC (WHO I > GBM), branch-point density (WHO I < GBM), and skeleton length density (WHO I < GBM).

Nonparametric Kruskal–Wallis tests revealed statistically significant overall group effects for skeleton branch-point density (H = 11.47, *p* = 0.0032), skeleton length density (H = 8.39, *p* = 0.0151), and radial-AUC (H = 7.31, *p* = 0.0258). No significant group effects were observed for entropy (H = 0.67, *p* = 0.716), tail-AUC (H = 3.47, *p* = 0.177), isoperimetric ratio (H = 4.66, *p* = 0.097), or convexity (H = 1.79, *p* = 0.408). These results are summarized in Table 2 and the distribution is visualized in Fig. 2.

Pairwise Mann–Whitney *U* tests followed by Benjamini–Hochberg false discovery rate (FDR) correction (q < 0.05) identified three significant contrasts:

1. Radial-AUC: WHO I > GBM (U = 109, *p* = 0.007, q = 0.049, cliff's δ = −0.68, Hodges–Lehmann shift = +0.46).

WHO I meningiomas exhibited greater radial persistence of instability relative to GBM.

2. Skeleton branch-point density: WHO I < GBM (U = 10, *p* = 7.3 × 10^−4^, q = 0.015, δ = +0.85, HL = −0.12).

GBMs showed a substantially higher density of branch points within unstable regions.

3. Skeleton length density: WHO I < GBM (U = 17, *p* = 0.0032, q = 0.034, δ = +0.74, HL = −0.18).

GBMs presented longer cumulative skeleton structures per area compared to WHO I lesions.

All Mann–Whitney U statistics, adjusted q-values, effect sizes, and Hodges–Lehmann median shifts are reported in Table 3.

### 3.4. Group-average spatial probability maps

**Fig. 3.**
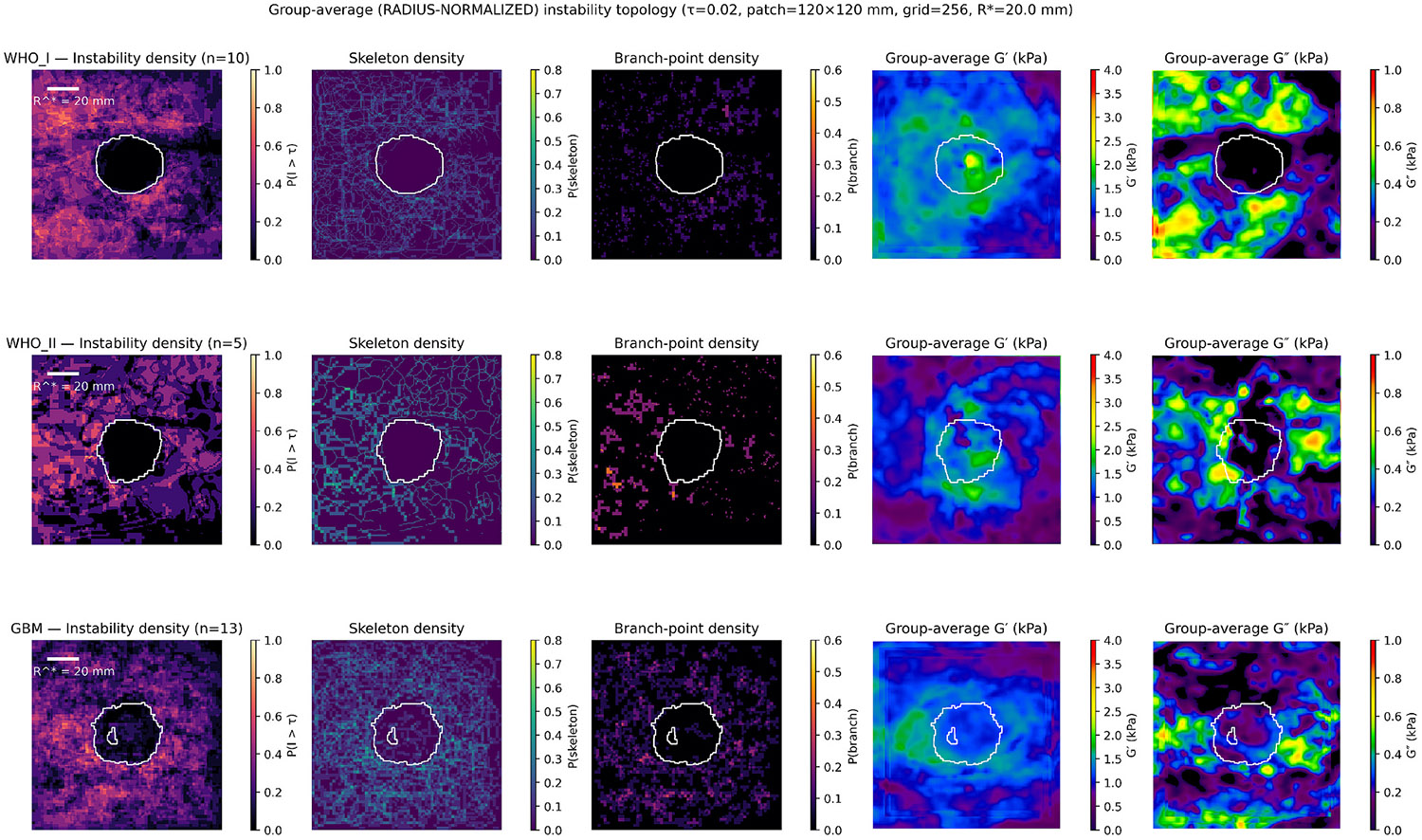
Group-average radius-normalized instability and skeleton maps in WHO I, WHO II, and glioblastoma. Group-average, radius-normalized maps of peritumoral mechanical instability and derived topological descriptors for WHO I meningiomas (top row), WHO II meningiomas (middle row), and glioblastomas (GBM; bottom row). Instability density maps show the probability that the instability index exceeds the threshold τ = 0.02 within a 120 × 120 mm patch centered on the tumor, after normalization to a common effective tumor radius (R* = 20 mm). Skeleton density and branch-point density maps summarize the spatial organization and topological complexity of suprathreshold instability regions, highlighting filamentous structures and their bifurcations. White contours indicate the group-average tumor boundary in normalized space. The rightmost panels show group-average storage modulus (G′) and loss modulus (G″), displayed in kPa, providing viscoelastic context for the observed instability patterns. Color scales are fixed across groups for comparability. Values near zero indicate mechanical equilibrium with the peritumoral reference state, whereas elevated instability reflects regions of dynamic viscoelastic mismatch at the tumor–brain interface.

Radius normalized group-average instability and skeleton probability maps are shown in Fig. 3 and non-normalized maps in S1.

Each heatmap represents the voxelwise average of binarized instability (I>0.02), skeleton, and branch-point maps across all subjects within each diagnostic group, resampled to a common 120 × 120 mm physical grid.

Instability occupancy was most spatially compact in WHO I meningiomas, showed irregular expansion in WHO II, and appeared diffuse and branched in GBMs.

Skeleton and branch-point probability increased progressively with malignancy, consistent with the quantitative metrics summarized above.

### 3.5. Robustness

Varying the instability threshold from 0.015 to 0.030 did not alter group ordering or qualitative patterns. GBMs consistently showed more diffuse peritumoral fields (lower radial-AUC than WHO I meningiomas). Effect-size differences across thresholds were small (< 10%), indicating that results were not driven by a specific cutoff. Bootstrap resampling showed limited robustness of WHO II ordering across instability topology metrics. For skeleton length density and branch-point density, the probability that WHO II medians lay between WHO I and GBM was 0.52 and 0.50, respectively, with 95% confidence intervals for WHO II–WHO I and GBM–WHO II median differences crossing zero in both metrics. For radial-AUC, WHO II did not exhibit intermediate ordering in any bootstrap iteration (*P* = 0.00); WHO II–WHO I median differences spanned zero (95% CI −1.31 to 0.51), while GBM–WHO II differences also crossed zero (95% CI −0.85 to 0.48).

Applying a light morphology opening before skeletonization changed median skeleton metrics by <8% and preserved group rankings, showing that filament and branch descriptors were not artifacts of pixel-scale noise.

Negative I(x) voxels accounted for 18 ± 7% across cases (WHO I ≈ 21%, GBM ≈ 15%) confirming that peritumoral behavior is largely dissipative. These values indicate more localized stability in meningiomas and a broader dissipative field in GBMs. Median radial-AUC increased slightly with threshold across all groups but maintained the same ordering (GBM < WHO II < WHO I). Radial-AUC showed only weak associations with tumor size proxies (∣ρ∣ < 0.25), confirming that group averages in physical space were not dominated by larger tumors.

Radius-normalized probability maps reproduced the same spatial organization observed in physical coordinates—compact and symmetric for WHO I, heterogeneous for WHO II, and diffuse and branched for GBM—demonstrating that the observed topology differences are intrinsic rather than scale-dependent (S1).

Stiffness contrast $\left(\Delta G^{\prime }\right)$ correlated inversely with radial-AUC (ρ = −0.46, *p* = 0.014), indicating that higher stiffness contrast is associated with a more compact, rim-confined instability field. Δtanδ showed no significant associations, and neither contrast predicted skeleton or branch-point densities (S2). N-maps are available in the supplementary material (S3) and visualize how many samples contributed to each voxel by group.

Together, these analyses confirm that group differences in instability topology—compact, radially coherent fields in WHO I meningiomas; broader, irregular fields in WHO II; and diffuse, fragmented fields in GBM—are robust to thresholding, morphological filtering, and size normalization. However the intermediate categorization of WHO II cannot be confirmed with the current sample size.

## 4. Discussion

This study establishes a quantitative framework for characterizing peritumoral mechanical instability from MRE and demonstrates distinct spatial organizations of viscoelastic imbalance across intracranial tumor types. By modeling instability as a voxelwise divergence between elastic storage and viscous dissipation, we show that benign and malignant tumors exhibit fundamentally different modes of mechanical coupling to the surrounding brain.

In WHO I meningiomas, instability fields were spatially compact, smooth, and radially coherent, with high radial-AUC and low skeleton densities. In contrast, GBMs displayed diffuse, branched instability networks with markedly higher skeleton length and branch-point densities and a rapid radial decay. WHO II meningiomas seem to occupy an intermediate position between these extremes. These findings indicate that tumors with higher invasive potential exhibit reduced mechanical coherence and greater viscoelastic dissipation at the brain interface.

### 4.1. Definition and interpretation of the instability index

The instability index was formulated as a relative measure of dynamic viscoelastic mismatch at the tumor–brain interface, rather than as an absolute descriptor of tissue stiffness or viscosity. It is defined as the product of a logarithmic contrast in viscous dissipation (G″) and a deviation in phase lag (tanδ), both referenced to the peritumoral baseline. The multiplicative structure enforces that elevated instability arises only when dissipation magnitude and phase behavior deviate concurrently, whereas isolated changes in either parameter alone yield low or subthreshold values. Positive instability values indicate co-directional deviations that reinforce dissipative imbalance under cyclic loading, while negative values reflect counter-directional viscoelastic changes that signify mechanical difference without dissipative runaway. Values near zero correspond to mechanical equilibrium with the local reference state. Accordingly, the instability index is a directional, interface-specific marker of viscoelastic imbalance rather than a monotonic measure of abnormality.

### 4.2. Mechanical coupling and interface coherence

The tumor–brain interface represents a boundary between two viscoelastic continua. When elastic and viscous stresses remain balanced, the interface deforms coherently; when this balance is disrupted, energy is dissipated through localized deformation or slip [21,23,24,29,30]. The confined instability patterns in meningiomas suggest that elastic storage dominates near the interface, preserving structural coherence and a predictable dissection plane. This elastic dominance corresponds to a mechanically stable configuration, consistent with high stiffness and low damping in MRE studies and with the absence of Saffman–Taylor-type instabilities. In contrast, glioblastomas, which exhibit lower stiffness and reduced viscous storage, fulfill the conditions for viscous fingering and display diffuse, branched instability fields consistent with interface decoupling and infiltrative behavior [31]. Conversely, the fragmented instability topology in GBM aligns with a more viscous, energy-dissipative microenvironment where coupling fails locally, analogous to branching seen in soft-matter systems under differential strain [4,20,23]. The mechanical transition observed here—from coherent to branched instability—may therefore represent a physical signature of viscoelastic decoupling during malignant invasion^32-34.^The mechanical instability framework is related to, but distinct from, prior work on tumor–brain interface adhesion and slip-interface imaging (SII) [32-35]. SII characterizes the local boundary condition at the tumor–brain interface, assessing whether relative motion is permitted or constrained using strain-based MRE signatures. he instability index quantifies the direction and spatial organization of viscoelastic contrast between tumor and adjacent brain tissue, capturing how differences in dissipation and phase behavior manifest in the peritumoral region under cyclic loading. In this formulation, interface adhesion governs how mechanical stress is transmitted across the boundary, whereas instability mapping reflects how that transmitted stress is accommodated and redistributed in surrounding parenchyma. These approaches therefore probe complementary layers of tumor–brain mechanical interaction, and their combined assessment may provide a more complete description of interface coupling and the resulting spatial organization and extent of peritumoral viscoelastic instability.

### 4.3. Topological metrics as descriptors of viscoelastic heterogeneity

Skeleton length and branch-point densities provided compact descriptors of the topology of mechanical instability. These parameters quantify the complexity of the unstable network rather than its amplitude, thereby capturing fragmentation of mechanical coherence. Skeletonization identifies the medial axes of contiguous instability regions and thus represents preferential pathways along which dissipative mismatch is spatially organized in the peritumoral tissue. Branch points mark locations where these pathways split or intersect, reflecting increased topological complexity and redistribution of mechanical load. From a physical perspective, higher skeleton density indicates more coherent or filamentous organization of viscoelastic imbalance, whereas increased branching indicates fragmentation of instability into multiple pathways. By reducing a high-dimensional voxelwise instability field to compact topological descriptors, skeleton and branch measures facilitate comparison of spatial organization across tumors and groups. Their progressive increase from WHO I to GBM parallels histological observations of irregular ECM architecture and variable adhesion at malignant interfaces [8,25]. Radial-AUC, conversely, describes the spatial persistence of instability away from the tumor margin, indicating the extent to which mechanical perturbations penetrate adjacent brain. Together, these metrics define complementary axes of interface mechanics: *coherence* (radial-AUC) and *fragmentation* (skeleton topology). Their combination offers a quantitative language for describing tumor–parenchyma coupling, analogous to stiffness and damping parameters but with spatial context.

### 4.4. Clinical and translational significance

Understanding how tumors mechanically interact with surrounding tissue has direct relevance for neurosurgical tumor resection and for emerging clinical elasticity-guided strategies [10,31]. Compact, coherent instability fields—as in low-grade meningiomas—imply predictable deformation behavior and a stable cleavage plane, whereas diffuse and branched patterns—as in GBM—suggest heterogeneous spread and potential for irregular motion or residual infiltration. Quantitative instability mapping could therefore complement conventional stiffness measures in preoperative assessment, predicting microinvasion, recurrence etc.

Local variations in the instability field may also reflect boundary conditions imposed by nearby anatomical structures such as ventricles or major white-matter tracts. These local constraints could influence how tumors deform or invade surrounding tissue, effectively channeling growth along paths of least mechanical resistance.

Beyond resection planning, instability topology provides a framework for linking in vivo mechanics to extracellular matrix remodeling and invasive potential [6,8,25]. When combined with diffusion imaging, radiomics, or molecular data, these maps could improve non-invasive tumor phenotyping and treatment stratification. Longitudinal monitoring of instability patterns may further enable early detection of biomechanical changes that precede radiographic progression or therapeutic response, and be used to predict the growth pattern [21,23,24]. Spatial patterns of instability might help explain where tumors tend to extend or recur after treatment. Such integrative, elasticity-informed approaches could ultimately refine surgical strategy, guide therapy, and support the development of mechanically targeted interventions in neuro-oncology.

### 4.5. Future directions

Future work should integrate instability mapping with histological, proteomic, and rheometric data to elucidate the biochemical substrates of mechanical fragmentation. Coupling instability fields with dynamic MRE at multiple drive frequencies may also reveal frequency-dependent bifurcation behavior and fluid–solid coupling in vivo. Longitudinal monitoring could determine whether changes in instability topology accompany treatment response or recurrence, potentially establishing a biomechanical biomarker of therapeutic effect and prediction of the growth pattern.

### 4.6. Methodological considerations, limitations and validation

The instability index reduces complex viscoelastic behavior to a scalar, interface-referenced measure of local imbalance. While this abstraction omits direction and anisotropic effects, it enables reproducible voxelwise comparisons. The spatial domain was selected to capture the immediate mechanical transition zone at the tumor–brain interface, although more extended fields may reveal longer-range poroelastic interactions with future 3D registration offering improved geometric fidelity. Although branched instability patterns were more prominent in GBM, their interpretation as markers of infiltrative growth remains inferential; direct voxelwise validation against histological invasion margins is possible, but limited by sparse sampling and loss of spatial correspondence due to brain shift and tissue deformation during resection. Importantly, instability mapping is intended as an in vivo, dynamic phenotype of tumor–brain mechanical interaction. Longitudinal imaging could provide the most appropriate validation framework. Future work should therefore emphasize imaging-based endpoints, including prediction of spatial growth or recurrence topology and detection of within-patient instability changes during treatment. In this context, multifrequency MRE represents a particularly promising extension, as frequency-dependent viscoelastic contrast would effectively introduce a temporal dimension at each voxel, enabling assessment of dispersion-related instability and its evolution over time. Finally, the moderate sample size limits detection of subtler differences, particularly between WHO I and II lesions, and validation in larger prospective cohorts is warranted.

## 5. Conclusions

Peritumoral mechanical instability mapping reveals systematic differences in the spatial organization of viscoelastic imbalance between WHO I- and WHO II meningiomas, and GBMs. Low-grade meningiomas exhibit confined, coherent, and radially ordered instability and may reflect stable elastic coupling, whereas GBMs show diffuse, branched, and fragmented patterns indicative of dissipative decoupling. These observations link macroscopic mechanical topology to tumor biology and operative and invasive behavior, suggesting that instability mapping may serve as a quantitative tool for assessing tumor–brain interface integrity and guiding elasticity-based strategies.

## Supplementary Material

1

## Figures and Tables

**Table 1:** Cohort characteristics.

Group	N	Age mean (range)	Male	Female	MGMT methylated	MGMT unmethylated	MGMT unknown
WHO I meningioma	10	63.6 (40–86)	4	6	–	–	–
WHO II meningioma	5	67.4 (58–80)	0	5	–	–	–
Glioblastoma	13	66.2 (46–86)	5	8	7	5	1

**Table 2:** Groupwise medians and Kruskal–Wallis test results for all instability-derived metrics.

Metric	WHO I Median	WHO II Median	GBM Median	H Statistic	*p* Value	Significance
Skeleton branch-point density	0.02	0.03	0.15	11.47	0	*
Skeleton length density	0.13	0.32	0.33	8.39	0.02	*
Radial-AUC	1.19	0.55	0.71	7.31	0.03	*
Isoperimetric ratio (IPR)	8.72	8.75	18.06	4.66	0.1	ns
Tail-AUC	0.14	0.1	0.11	3.47	0.18	ns
Convexity	0.54	0.54	0.5	1.79	0.41	ns
Entropy	4.02	3.88	3.94	0.67	0.72	

**Table 3:** Pairwise Mann–Whitney U comparisons with Benjamini–Hochberg FDR correction.

Metric	Groups Compared	U Statistic	p Value	q (FDR)	Cliff's δ	Hodges–Lehmann Shift	Significance
Radial-AUC	WHO I > GBM	109	0.01	0.05	−0.68	0.46	*
Skeleton branch-point density	WHO I < GBM	10	7.3 × 10^−4^	0.02	0.85	−0.12	*
Skeleton length density	WHO I < GBM	17	0	0.03	0.74	−0.18	*
Radial-AUC	WHO I vs WHO II	37	0.17	0.43	−0.48	0.46	ns
Radial-AUC	WHO II vs GBM	34	0.92	0.95	−0.05	0.02	ns
Skeleton branch-point density	WHO I vs WHO II	20.5	0.62	0.83	0.18	−0.01	ns
Skeleton branch-point density	WHO II vs GBM	16	0.12	0.39	0.51	−0.09	ns
Skeleton length density	WHO I vs WHO II	14.5	0.22	0.43	0.42	−0.10	ns
Skeleton length density	WHO II vs GBM	27	0.63	0.83	0.17	−0.02	ns
Entropy	WHO I vs GBM	79	0.4	0.65	−0.22	0.09	ns
Convexity	WHO I vs GBM	86	0.2	0.43	−0.32	0.06	ns
IPR	WHO I vs GBM	37	0.09	0.37	0.43	−7.00	ns

## Data Availability

Data will be made available on request.

## Statement of significance

Intracranial tumors can disrupt the mechanical equilibrium of the brain, yet how these changes govern tissue deformation, invasion, and surgical behavior remains unclear. Here we introduce *mechanical instability mapping*—a framework that quantifies the spatial organization of viscoelastic imbalance around tumors using magnetic resonance elastography. Distinct topological patterns of instability differentiate benign from malignant lesions: meningiomas preserve coherent elastic coupling, whereas glioblastomas display fragmented, dissipative fields indicative of invasive, mechanically decoupled growth. These findings identify a new biomechanical signature of tumor–brain interaction and establish instability topology as a quantitative link between imaging, histopathology, and operative mechanics, with potential applications in elasticity-guided neurosurgery, biomechanical phenotyping, and MR-based histopathology.

## Appendix A. Supplementary data

Supplementary data to this article can be found online at https://doi.org/10.1016/j.bioadv.2026.214758.

## References

[1] Budday S, Ovaert TC, Holzapfel GA, Steinmann P, Kuhl E, Fifty shades of brain: a review on the mechanical testing and modeling of brain tissue, Arch. Comput. Methods Eng 27 (2020) 1187–1230.

[2] Budday S, , Mechanical characterization of human brain tissue, Acta Biomater. 48 (2017) 319–340. 27989920 10.1016/j.actbio.2016.10.036

[3] Rashid B, Destrade M, Gilchrist MD, Mechanical characterization of brain tissue in simple shear at dynamic strain rates, J. Mech. Behav. Biomed. Mater 28 (2013) 71–85. 23973615 10.1016/j.jmbbm.2013.07.017

[4] Chaudhuri PK, Low BC, Lim CT, Mechanobiology of tumor growth, Chem. Rev 118 (2018) 6499–6515. 29927236 10.1021/acs.chemrev.8b00042

[5] Mierke CT, Viscoelasticity acts as a marker for tumor extracellular matrix characteristics, Front. Cell Dev. Biol 9 (2021) 785138. 34950661 10.3389/fcell.2021.785138 PMC8691700

[6] Franze K, Janmey PA, Guck J, Mechanics in neuronal development and repair, Annu. Rev. Biomed. Eng 15 (2013) 227–251. 23642242 10.1146/annurev-bioeng-071811-150045

[7] Paszek MJ, , Tensional homeostasis and the malignant phenotype, Cancer Cell 8 (2005) 241–254. 16169468 10.1016/j.ccr.2005.08.010

[8] Pogoda K, , Compression stiffening of brain and its effect on mechanosensing by glioma cells, New J. Phys 16 (2014) 075002. 10.1088/1367-2630/16/7/075002 PMC4380293 25844043

[9] Zada G, , A proposed grading system for standardizing tumor consistency of intracranial meningiomas, Neurosurg. Focus 35 (2013) E1. 10.3171/2013.8.FOCUS13274 24289117

[10] Duhon BH, , Tumor biomechanical stiffness by magnetic resonance elastography predicts surgical outcomes and identifies biomarkers in vestibular schwannoma and meningioma, Sci. Rep 14 (2024) 14561. 38914647 10.1038/s41598-024-64597-1 PMC11196577

[11] Černý M, , Utility of texture analysis for objective quantitative ex vivo assessment of meningioma consistency: method proposal and validation, Acta Neurochir. 165 (2023) 4203–4211. 38044374 10.1007/s00701-023-05867-1

[12] Itamura K, , Prospective clinical validation of a meningioma consistency grading scheme: association with surgical outcomes and extent of tumor resection, J. Neurosurg 131 (2019) 1356–1360. 30554187 10.3171/2018.7.JNS1838

[13] Shah I, , Association between meningioma consistency and surgical outcomes, J. Neurosurg 142 (2025) 1331–1337. 39793013 10.3171/2024.8.JNS241066

[14] Aunan-Diop JS, Friismose AI, Halle B, , Repeatability of magnetic resonance elastography-derived mechanical parameters in intracranial meningiomas, J. Magn. Reson. Imaging 62 (4) (2025) 1047–1058, 10.1002/jmri.29825. 40372143 PMC12353909

[15] Friismose AI, Aunan-Diop JS, Pedersen M, , Evaluating measurement stability in glioblastomas using magnetic resonance elastography: repeatability and interobserver agreement, J. Magn. Reson. Imaging (2025) 1–9, 10.1002/jmri.70145. 41061165

[16] Reiss-Zimmermann M, , High resolution imaging of viscoelastic properties of intracranial tumours by multi-frequency magnetic resonance elastography, Clin. Neuroradiol. 25 (2015) 371–378. 24916129 10.1007/s00062-014-0311-9

[17] Streitberger KJ, , How tissue fluidity influences brain tumor progression, Proc. Natl. Acad. Sci. USA 117 (2020) 128–134. 31843897 10.1073/pnas.1913511116 PMC6955323

[18] Streitberger KJ, , High-resolution mechanical imaging of glioblastoma by multifrequency magnetic resonance elastography, PLoS One 9 (2014). 10.1371/journal.pone.0110588 PMC4206430 25338072

[19] Dervaux J, Ben Amar M, Morphogenesis of growing soft tissues, Phys. Rev. Lett 101 (2008) 068101. 18764507 10.1103/PhysRevLett.101.068101

[20] Lawless J, Juel A, Pihler-Puzović D, Nonlinear dynamics of viscous fingering, Physica D 476 (2025) 134631.

[21] Mora S, Phou T, Fromental J-M, Pomeau Y, Gravity driven instability in elastic solid layers, Phys. Rev. Lett 113 (2014) 178301. 25379940 10.1103/PhysRevLett.113.178301

[22] Maher JV, Development of viscous fingering patterns, Phys. Rev. Lett 54 (1985) 1498–1501. 10031054 10.1103/PhysRevLett.54.1498

[23] Saffman PG, Taylor G, The penetration of a fluid into a porous medium or hele-Shaw cell containing a more viscous liquid, Proc. R. Soc. Lond. A Math. Phys. Sci 245 (1958) 312–329.

[24] Biot MA, Surface instability of rubber in compression, Appl. Sci. Res. Sect. A 12 (1963) 168–182.

[25] Stylianopoulos T, , Causes, consequences, and remedies for growth-induced solid stress in murine and human tumors, Proc. Natl. Acad. Sci. USA 109 (2012) 15101–15108. 22932871 10.1073/pnas.1213353109 PMC3458380

[26] Yin Z, , In vivo characterization of 3D skull and brain motion during dynamic head vibration using magnetic resonance elastography, Magn. Reson. Med 80 (2018) 2573–2585. 29774594 10.1002/mrm.27347 PMC6240411

[27] Fedorov A, , 3D slicer as an image computing platform for the quantitative imaging network, Magn. Reson. Imaging 30 (2012) 1323–1341. 22770690 10.1016/j.mri.2012.05.001 PMC3466397

[28] Hoopes A, Mora JS, Dalca AV, Fischl B, Hoffmann M, SynthStrip: skull-stripping for any brain image, NeuroImage 260 (2022) 119474. 35842095 10.1016/j.neuroimage.2022.119474 PMC9465771

[29] Keil VC, , DCE-MRI in glioma, infiltration zone and healthy brain to assess angiogenesis: a biopsy study, Clin. Neuroradiol 31 (2021) 1049–1058. 33900414 10.1007/s00062-021-01015-3 PMC8648693

[30] Whitfield GA, Kennedy SR, Djoukhadar IK, Jackson A, Imaging and target volume delineation in glioma, Clin. Oncol 26 (2014) 364–376. 10.1016/j.clon.2014.04.026 24824451

[31] Aunan-Diop JS, , Magnetic resonance Elastography in intracranial neoplasms: a scoping review, Top. Magn. Reson. Imaging 31 (2022) 9–22. 10.1097/RMR.0000000000000292 35225840

[32] Yin Z, , Slip Interface imaging predicts tumor-brain adhesion in vestibular schwannomas, Radiology 277 (2015) 507–517. 26247776 10.1148/radiol.2015151075 PMC4618713

[33] Yin Z, , Slip interface imaging based on MR-elastography preoperatively predicts meningioma-brain adhesion, J. Magn. Reson. Imaging 46 (2017) 1007–1016. 28194925 10.1002/jmri.25623 PMC5600107

[34] Zheng K, , Improved quantification of tumor adhesion in meningiomas using MR elastography-based slip interface imaging, PLoS One 19 (2024) e0305247. 38917107 10.1371/journal.pone.0305247 PMC11198761

[35] Hughes JD, , Prospective study of slip-interface imaging in meningioma for brain-tumor adhesion, J. Neurol. Surg. B Skull Base 78 (2017). Conference: 27th Annual Meeting North American Skull Base Society. United States.

