# Supplementary material for: Mechanical instability as a signature of viscoelastic decoupling at the tumor–brain interface
Source: Biomater Adv. Author manuscript; Available in PMC 2026 Mar 23. (PMC13007089; doi:10.1016/j.bioadv.2026.214758)
Supplement: 1 [file NIHMS2156225-supplement-1.docx]

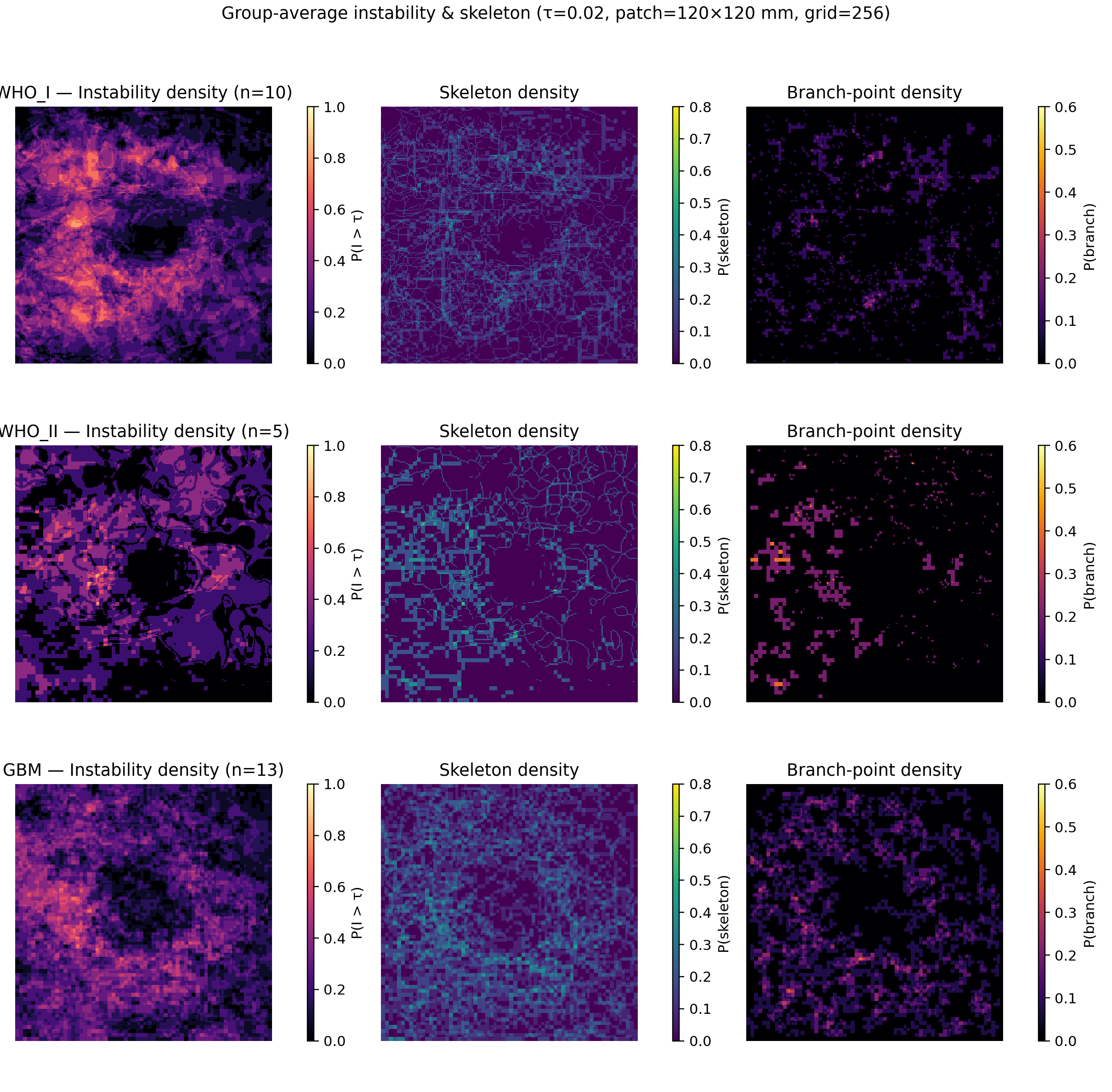
***Supplementary Figure S1. Group average non-normalized instability maps***

###
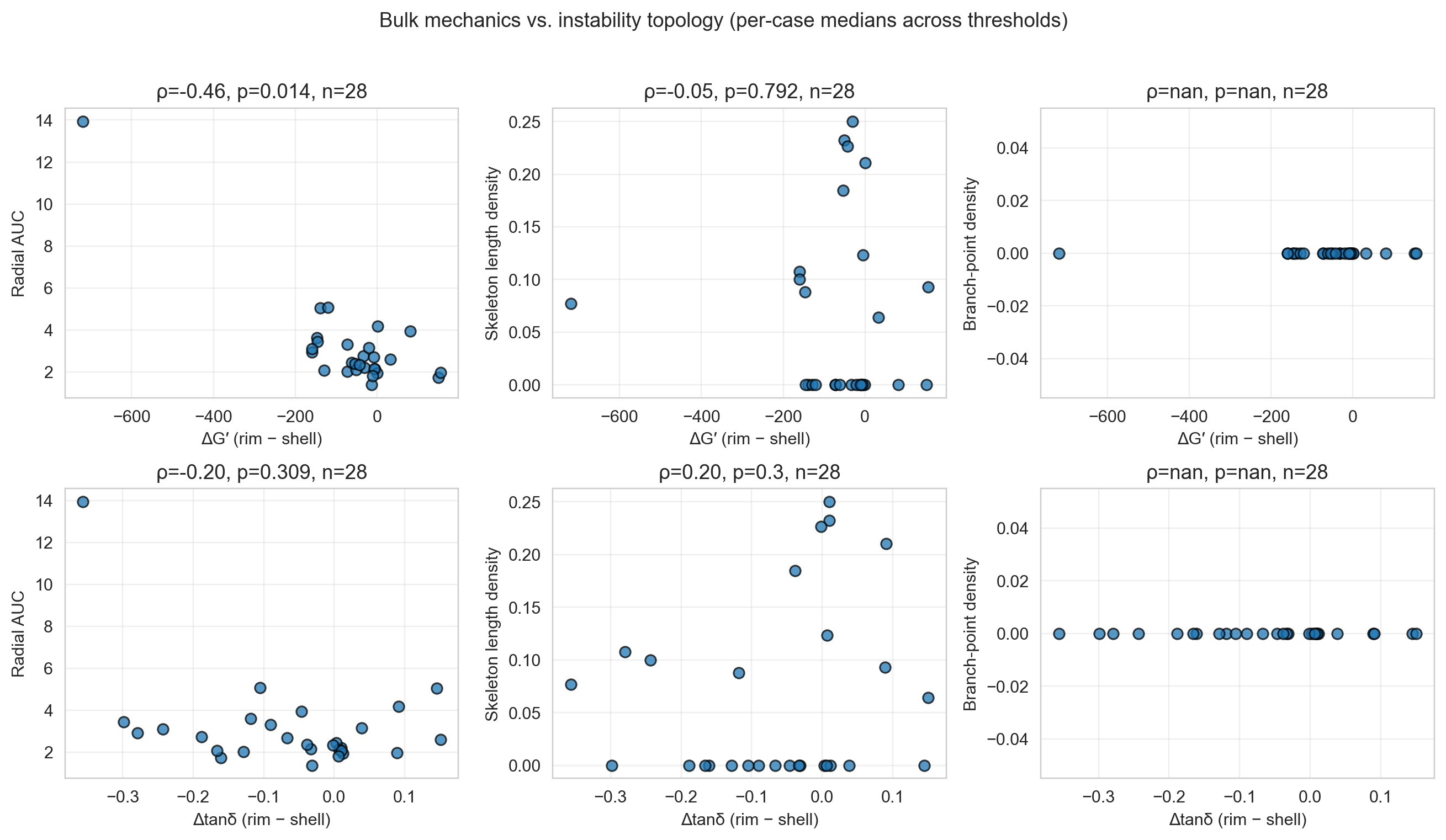
*Supplementary Figure S2. Relationship between bulk mechanics and instability topology*

*Scatter-matrix showing correlations between rim–shell mechanical contrasts—ΔG′ = G′₍rim₎ – G′₍shell₎ and Δtan δ = tan δ₍rim₎ – tan δ₍shell₎—and topology metrics (radial-AUC, skeleton length density, branch-point density). Each panel reports the Spearman correlation coefficient (ρ), p-value, and number of cases (n = 28). A significant inverse relationship was observed between ΔG′ and radial-AUC (ρ = –0.46, p = 0.014), indicating that higher stiffness contrast corresponds to more compact instability fields. No other associations reached significance.*

###
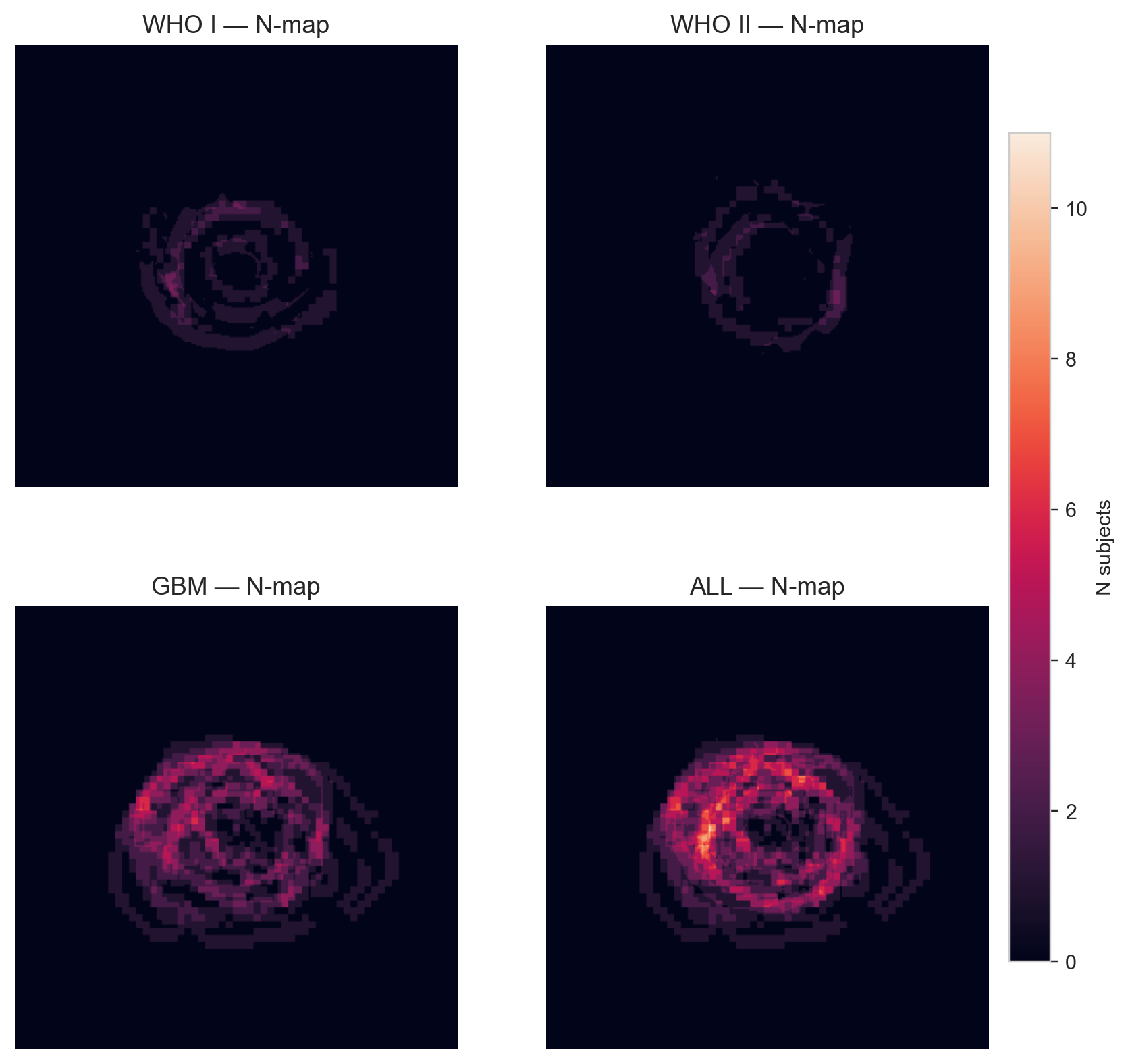
*Supplementary Figure S3. Group coverage (N-maps):*

*Coverage maps showing the number of contributing subjects (N) per voxel after spatial normalization for WHO I, WHO II, GBM, and combined (ALL) cohorts. The maps visualize how many cases contained valid peritumoral tissue at each pixel, providing context for group-average reliability. Higher N values in the GBM and ALL maps reflect their larger sample sizes and broader peritumoral coverage.*
